# A variant in *IL6ST* with a selective IL-11 signaling defect in human and mouse

**DOI:** 10.1038/s41413-020-0098-z

**Published:** 2020-06-11

**Authors:** Tobias Schwerd, Freia Krause, Stephen R. F. Twigg, Dominik Aschenbrenner, Yin-Huai Chen, Uwe Borgmeyer, Miryam Müller, Santiago Manrique, Neele Schumacher, Steven A. Wall, Jonathan Jung, Timo Damm, Claus-Christian Glüer, Jürgen Scheller, Stefan Rose-John, E. Yvonne Jones, Arian Laurence, Andrew O. M. Wilkie, Dirk Schmidt-Arras, Holm H. Uhlig

**Affiliations:** 1 Translational Gastroenterology Unit, John Radcliffe Hospital, University of Oxford, Oxford, UK; 2 Department of Pediatrics, Dr von Hauner Children’s Hospital, LMU Munich, Munich, Germany; 3 Christian-Albrechts-University Kiel, Institute of Biochemistry, Kiel, Germany; 4 Clinical Genetics Group, MRC Weatherall Institute of Molecular Medicine, University of Oxford, John Radcliffe Hospital, Oxford, UK; 5 Center for Molecular Neurobiology Hamburg (ZMNH), University Medical Center Hamburg-Eppendorf, Hamburg, Germany; 6 Division of Structural Biology, Wellcome Trust Centre for Human Genetics, University of Oxford, Oxford, UK; 7 Craniofacial Unit, Department of Plastic and Reconstructive Surgery, Oxford University Hospitals NHS Foundation Trust, John Radcliffe Hospital, Oxford, UK; 8 Section Biomedical Imaging, Department of Radiology and Neuroradiology, University Medical Center Schleswig-Holstein, Kiel, Germany; 9 Institute of Biochemistry and Molecular Biology II, Medical Faculty, Heinrich-Heine-University, Düsseldorf, Germany; 10 Department of Paediatrics, University of Oxford, Oxford, UK; 11 NIHR Oxford Biomedical Research Centre, Oxford, UK; 12 Present Address: The Beatson Institute for Cancer Research, Glasgow, UK; 13 Present Address: School of Medicine, University of Glasgow, Glasgow, UK

**Keywords:** Bone, Pathogenesis

## Abstract

The GP130 cytokine receptor subunit encoded by *IL6ST* is the shared receptor for ten cytokines of the IL-6 family. We describe a homozygous non-synonymous variant in *IL6ST* (p.R281Q) in a patient with craniosynostosis and retained deciduous teeth. We characterize the impact of the variant on cytokine signaling in vitro using transfected cell lines as well as primary patient-derived cells and support these findings using a mouse model with the corresponding genome-edited variant *Il6st* p.R279Q. We show that human GP130 p.R281Q is associated with selective loss of IL-11 signaling without affecting IL-6, IL-27, OSM, LIF, CT1, CLC, and CNTF signaling. In mice *Il6st* p.R279Q lowers litter size and causes facial synostosis and teeth abnormalities. The effect on IL-11 signaling caused by the GP130 variant shows incomplete penetrance but phenocopies aspects of *IL11RA* deficiency in humans and mice. Our data show that a genetic variant in a pleiotropic cytokine receptor can have remarkably selective defects.

## Introduction

GP130 is the common receptor subunit for the family of interleukin (IL)-6 cytokines that includes IL-6, IL-11, IL-27, leukemia inhibitory factor (LIF), oncostatin M (OSM), ciliary neurotrophic factor (CNTF), cardiotrophin 1 (CT1), and cardiotrophin-like cytokine (CLC).^1^ To allow specificity of cytokine receptor binding in different cell types, GP130 forms a multimeric complex on the cell surface with cytokine-selective receptor subunits that are either signaling-incompetent such as IL6RA, IL11RA, CNTFR, or signaling competent such as LIFR, OSMR, or IL-27R facilitating downstream activation of cytoplasmic tyrosine kinases which then phosphorylate STAT3 and STAT1 or activate the RAS/MAPK pathway.^1^

Diverse biologic functions of immune and non-immune cells depend on GP130-mediated signals. For example, bone formation and remodeling require IL-11 signaling through the GP130/IL11RA receptor complex. In mice, the absence of GP130 leads to skeletal abnormalities associated with defects in osteoblast and osteoclast function^2,3^ and osteoblast-specific disruption of GP130-STAT3 pathway impairs bone formation.^4^ Similarly, IL-11 receptor knockout (*Il11ra*^*−/−*^*)* mice show increased trabecular bone volume and synostosis of premaxillary sutures associated with a cell-autonomous defect of osteoclast differentiation.^5,6^

Genetic defects in genes required for IL11RA-dependent STAT3 signaling cause skeletal abnormalities in humans. Skeletal and connective tissue abnormalities are commonly found in patients with autosomal dominant hyper-IgE syndrome (HIES) due to heterozygous mutations in STAT3, including craniosynostosis, varying degrees of scoliosis or retained primary teeth.^7–13^ Patients with craniosynostosis and dental anomalies (CRSDA; MIM 614188) were found to carry recessive loss-of-function variants of *IL11RA*.^5,14–18^ The clinical disease phenotype is characterized by multi-suture craniosynostosis, maxillary hypoplasia, delayed or ectopic tooth eruption, supernumerary teeth and minor digit abnormalities. Craniosynostosis and delayed tooth eruption observed in individuals with *IL11RA* mutations, likely result from reduced bone resorption at sutures or in the jaw, as it has been shown that IL11RA-deficient mice display decreased bone resorption in long bones.^6^ We have recently described patients with severe immunodeficiency and skeletal abnormalities, such as severe craniosynostosis and progressive scoliosis caused by recessive partial loss-of-function variants in *IL6ST*, encoding GP130 (hyper-IgE recurrent infection syndrome 4, autosomal recessive; MIM 618523).^19,20^ Detailed functional studies demonstrated that different homozygous non-synonymous variants had relatively selective effects on the broad range of GP130-dependent cytokines with complete abrogation of IL-6 and IL-11 signals, reduction in OSM and IL-27 signaling but preserved LIF signaling. Similarly, autosomal-dominant variants in the cytoplasmic tail of GP130 cause a hyper-IgE recurrent infection syndrome due to defective IL-6 and IL-11 signaling (Beziat V., et al., *JEM* 2020 in press). Complete abrogation of all GP130-dependent cytokine signaling due to biallelic essential loss-of-function variants in GP130 causes an extended Stüve–Wiedemann syndrome with neonatal lethality.^21^

Here, we describe a patient with a biallelic, non-synonymous variant in GP130 with a highly selective defect limited to IL-11 signal transduction. The patient’s phenotype was restricted to craniosynostosis and tooth abnormalities. We demonstrate that the variant has incomplete penetrance in humans and mice, suggesting a hypomorphic modifier effect.

## Results

### *IL6ST* p.R281Q in a patient with craniosynostosis

**Fig. 1:**
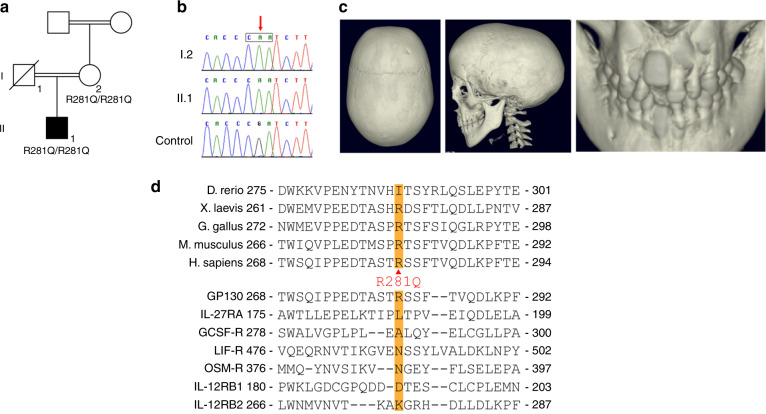
Identification of homozygous p.R281Q variant in a patient with craniosynostosis. **a** Pedigree of patient P^R281Q^ (II.1) showing segregation of *IL6ST* alleles. Note that both P^R281Q^ and his mother (I.2) are homozygous for the p.R281Q substitution. DNA of the father was not available. **b** Dideoxy-sequencing of the P^R281Q^ family, showing homozygosity for the c.842G>A variant. **c** Reconstructed CT scan of head of P^R281Q^ shows mild sagittal and bilambdoid synostosis and supernumerary teeth at the age of 9 years. **d** Alignment of GP130 protein sequence around the amino acid position p.R281 (top panel, multiple species alignment; bottom panel, cytokine receptor alignment). Substitution to glutamine (Q) is indicated between the panels. Note that amino acid R281 is evolutionarily conserved from amphibian to mammals but not conserved across receptors of the GP130 family

We recently described loss-of-function variants in *IL6ST* as a novel cause of autosomal recessive HIES with skeletal abnormalities.^19,20^ In order to understand the impact of *IL6ST* variants in patients with skeletal abnormalities, we screened for homozygous or compound heterozygous variants in *IL6ST* in a cohort of 467 unrelated patients with craniosynostosis, who were mutation negative after clinically driven genetic testing.^19^ We identified a homozygous variant (c.842G>A; p.R281Q) in a single patient of South Asian origin, hereafter referred to as P^R281Q^ (Fig. 1a–c). This individual presented at the age of 7 years with abnormal head shape associated with sagittal and bilateral lambdoid craniosynostosis and retained deciduous teeth, reportedly requiring the extraction of 14 teeth aged 8 years. Intracranial pressure monitoring was normal and on annual follow-up no clinical progression requiring surgical intervention for the craniosynostosis was observed. There were no infections or immune dysregulation problems up to the age of 18 years. Clinical genetic testing had been negative for the major causes of craniosynostosis, including *ERF, FGFR1 exon 7, FGFR2* (all exons associated with craniosynostosis), *FGFR3* exons 7 and 10, *IL11RA, TCF12* and *TWIST1*.

The amino acid R281 is conserved throughout evolution from amphibians to mammals (Fig. 1d, Supplementary Fig. 3a). The mutational impact of the p.R281Q substitution is predicted to be moderate; SIFT tolerated (0.162), PROVEAN neutral (−0.81), PolyPhen2 probably damaging (0.999), and with a CADD score 9.782.

We initially classified this as a variant of unknown significance since: (a) 48 (updated 20/11/2019) heterozygous individuals (but no homozygotes) are tabulated in gnomAD v3, (b) this variant is enriched in individuals of South Asian origin (minor allele frequency 0.001 5), (c) the mother of P^R281Q^, herself the offspring of consanguineous parents, was homozygous for the same variant (Fig. 1a) but without any history of craniofacial or severe tooth abnormalities, and (d) the phenotype was only partially overlapping with the previously described humans with *IL6ST* defects (Supplementary Table 1).

### p.R281Q causes defective IL-11 signal transduction while maintaining normal signaling of other IL-6 family cytokines

**Fig. 2:**
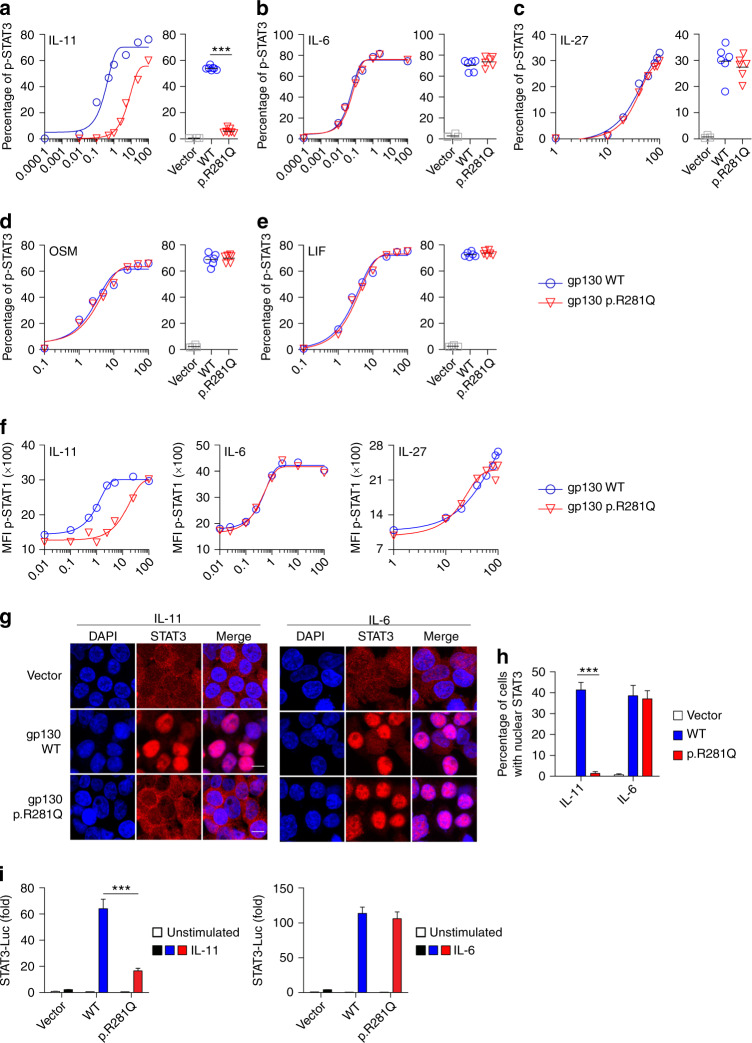
The GP130 p.R281Q substitution causes defective signaling of IL-11, but not IL-6, IL-27, OSM, and LIF. **a–e** HEK293 GP130-KO cells were transfected with empty vector control or plasmids encoding GP130 wild type (WT) or the patient variant p.R281Q. Cells were stimulated with indicated concentrations of IL-11 (**a**), IL-6 (**b**), IL-27 (**c**), OSM (**d**), or LIF (**e**) for 15 min and analyzed for STAT3 phosphorylation (pSTAT3) by phosflow. For assessment of IL-11 and IL-6 signaling, cells were co-transfected with plasmids encoding IL11RA and IL6RA, respectively. Co-transfection with GFP allowed gating on successfully transfected cells. Representative titration curves (on left in each panel) are shown for each ligand and are representative of two independent experiments. Curve fitted by non-linear regression. Quantification (on right in each panel) is based on 4–6 independent experiments per cytokine at one concentration (IL-11 1 ng·mL^−1^; IL-6, IL-27, OSM, LIF all 100 ng·mL^−1^). **f** Experiments with HEK293 GP130-KO cells performed as in **a**–**c**. Cells were assayed for phospho-STAT1 (pSTAT1). Titration curves are representative of two independent experiments. **g** Immunofluorescence staining of HEK293 GP130-KO cells, plated in chamber slides and transfected as in **a**. Cells were stimulated with 1 ng·mL^−1^ IL-11 (left) or 0.5 ng·mL^−1^ IL-6 (right) and analyzed for STAT3 nuclear translocation using confocal microscopy. Bars mark 10 μm. Images are representative for three independent experiments. **h** Quantification of **g**. At least 100 cells per experimental condition were quantified from three independent experiments each. Data represent mean ± s.e.m. **i** HEK293 GP130-KO cells were co-transfected with luciferase reporters, GP130 variant p.R281Q, and IL11RA or IL6RA-expression vectors, respectively. After 24 h, cells were stimulated with 1 ng·mL^−1^ IL-11 (left) or 0.5 ng·mL^−1^ IL-6 (right) for 6 h and induction of STAT3 reporter (relative to constitutively expressed Renilla luciferase) was determined. Results are expressed as fold-induction compared to unstimulated vector control and are pooled data from three independent experiments with 3–6 technical replicates. Data represent mean with SEM. Differences were investigated by Mann–Whitney *U* test (***P < 0.001)

To fully assess the functional consequences of the p.R281Q substitution, we used a previously described GP130-deficient HEK293 cell line (HEK293 GP130-KO) generated by CRISPR/Cas9 technology.^19^ This cell line does not phosphorylate STAT1 or STAT3 in response to stimulation with IL-6, IL-11, IL-27, OSM, or LIF, but has normal STAT3 signaling in response to type 1 interferon and normal STAT1 signaling in response to IFN-γ. Transfection with GP130 wild type (WT) restored GP130-dependent signaling (Fig. 2). p.R281Q did not confer mRNA or protein instability (data not shown). Titration studies on transfected GP130-KO cells revealed that the p.R281Q substitution significantly impaired STAT3 phosphorylation in response to IL-11 stimulation (Fig. 2a), but had little effect on IL-6, IL-27, OSM, or LIF induced STAT3 phosphorylation (Fig. 2b–e), as well as CT1, CLC, or CNTF induced STAT3 phosphorylation (Supplementary Fig. 1a–c). The p.R281Q substitution failed to rescue IL-11-induced STAT1 phosphorylation in a similarly selective manner (Fig. 2f).

Next, we used confocal microscopy to assess the functional consequences of p.R281Q mutated GP130 on IL-11-mediated STAT3 nuclear translocation. In HEK293 GP130-KO cells transfected with p.R281Q, the impaired STAT3 phosphorylation in response to IL-11 was associated with defective cytoplasmic to nuclear STAT3 translocation (Fig. 2g, h). Furthermore, we investigated the functional consequences of GP130 p.R281Q substitution in a STAT3 luciferase reporter assay. p.R281Q caused defective luciferase induction after IL-11 stimulation, whereas the IL-6-dependent STAT3 gene expression was not affected (Fig. 2i).

### IL-11 signaling defect in primary patient cells with endogenous GP130 p.R281Q

**Fig. 3:**
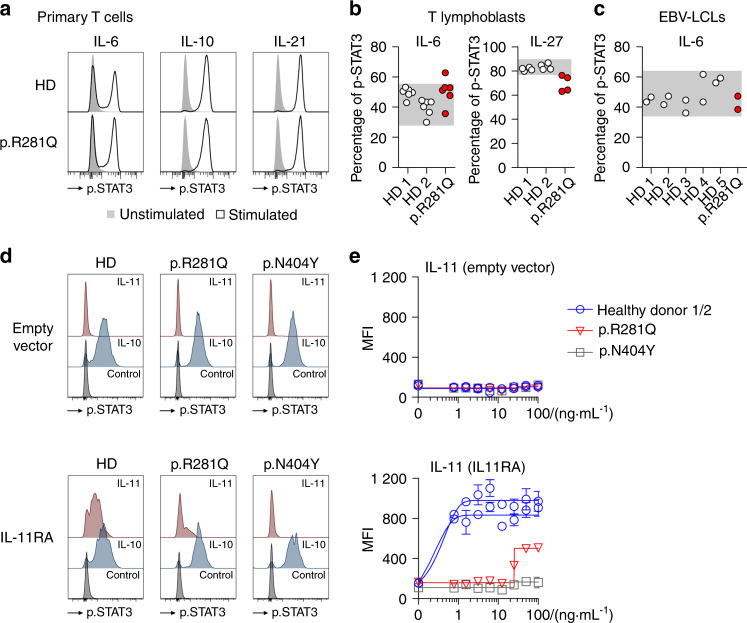
Ectopic expression of IL11RA with endogenous p.R281Q GP130 in primary CD4+ T cells confirms IL-11 signaling defect. **a** STAT3 phosphorylation is not altered in primary p.R281Q patient T cells compared to healthy donors (HD, one HD is shown). **b** Quantification of pSTAT3 T lymphoblasts from HDs and patient P^R281Q^ after stimulation with IL-6 and IL-27. **c** Analysis of pSTAT3 in lymphoblastoid cell lines (LCLs) following stimulation with IL-6. Experiments in **b** and **c** were performed in parallel to evaluation of patient P^N404Y^ described in Schwerd et al.^19^ and results from HDs are duplicated. Data represent pooled summary result from at least two independent experiments with 1–4 replicates. Phospho-STAT3 cells are determined based on unstimulated condition. Gray area indicates normal range based on healthy donors. **d**, **e** Lentiviral transduction and ectopic expression of an empty vector (top panel) or an IL11RA cDNA containing vector (bottom panel) in primary CD4^+^ T cells of two healthy donors (HD), patient P^R281Q^ and the previously described patient P^N404Y^. Results are shown from GFP^+^ gated populations. IL11RA-expressing T cells phosphorylate STAT3 in response to IL-11 stimulation in the context of endogenous GP130 expression. IL-10-induced STAT3 phosphorylation serves as GP130-independent control. **d** Representative histograms of two independent experiments and 3–5 replicates showing pSTAT3 level following 30 min stimulation with IL-10 or IL-11 (both 100 ng·mL^−1^). **e** Titration curves are representative of two independent experiments and 3–5 replicates. Curves are fitted by non-linear regression analysis. MFI mean fluorescence intensity

We next investigated GP130-dependent cytokines on primary T and B cells but did not find a defect in IL-6 signaling (Fig. 3a). We confirmed this finding with primary patient P^R281Q^-derived T lymphoblasts and an EBV-transformed lymphoblastoid cell line and found normal IL-27 signaling using T lymphoblasts (Fig. 3b, c).

Primary hematopoietic cells do not express the IL11RA and the individual P^R281Q^ declined a skin biopsy to establish fibroblast lines for IL-11 signaling studies. To overcome this problem and to measure IL-11 signaling in the available primary cells that endogenously express GP130, we transduced CD4^+^ lymphocytes of the patient with a lentivirus that encodes human IL11RA and made those cells responsive to IL-11. We used co-expression of GFP to control for unequal transduction efficiencies (Supplementary Fig. 2a, b). In contrast to primary CD4^+^ T cells that do not respond to IL-11, T cells transduced with WT GP130 became IL-11 responsive, whereas patient cells with the p.R281Q variant showed impaired responsiveness to IL-11 (Fig. 3d, e, Supplementary Fig. 2c). We used GP130 p.N404Y expressing primary T cells as control, as these cells do not respond to either IL-6 or IL-11 stimulation.^19^ This confirmed the selective IL-11 signaling defect caused by GP130 p.R281Q variant in primary patient cells.

### Homology modeling of the GP130/IL11RA/IL-11 complex provides insight into the cytokine-selective effects of GP130 p.R281Q

**Fig. 4:**
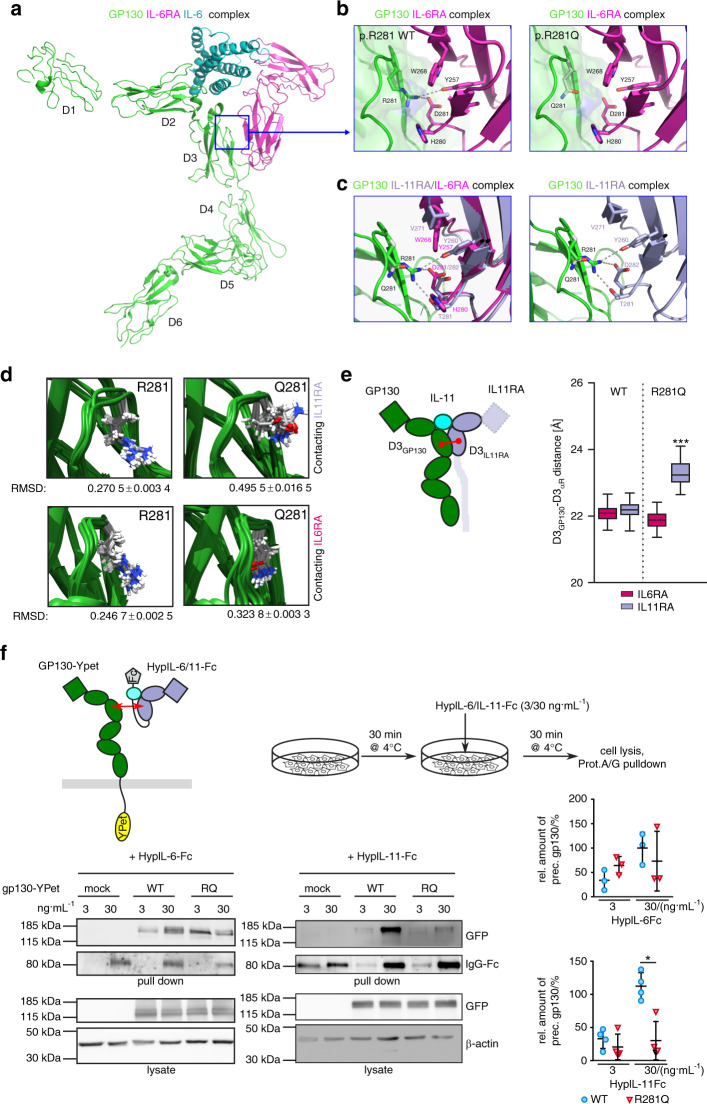
The R281Q substitution destabilizes the GP130/IL11RA/IL-11 but not the GP130/IL6RA/IL-6 receptor complex. **a** 3D representation of the GP130/IL6RA/IL-6 receptor complex (1P9M.pdb) which served as a starting point for the homology modeling of a GP130/IL11RA/IL-11 complex. **b** Close-up views of GP130/IL6RA/IL-6 complex WT(left) and R281Q model (right). **c** Close-up view of superimposed IL6RA on GP130/IL11RA homology model (left) and GP130/IL11RA homology model (right) showing disruption of hydrogen bonds and salt bridges due to GP130 p.R281Q substitution, affecting interactions with IL11RA Y260, T281, and D282. **d** Reduced interaction of GP130 Q281 to IL11RA but not IL6RA. Rotamer flexibility of the indicated residues during molecular dynamics (MD) simulation of the indicated GP130/α-receptor/cytokine complexes. Increased root mean square deviation (RMSD) of Q281 in the GP130/IL11RA/IL-11 complex indicates elevated flexibility due to impaired molecular interactions towards the IL11RA. Snapshots during MD simulation were taken every 0.1 ns and backbone α atoms were superimposed using the Matchmaker tool in the Chimera package. **e** Stability of D3–D3 interaction is weakened in the GP130 R281Q/IL11RA/IL-11 but not the GP130 R281Q/IL6RA/IL-6 complex. Mean center of mass D3_GP130_-D3_IL11RA_ and D3_GP130_-D3_IL6RA_ interdomain distance during MD simulation within a GP130/α-receptor/cytokine complex. *n* = 500 frames, ***P < 0.005, Mann–Whitney *U*. **f** Affinity of the IL-11/IL11RA complex but not the IL-6/IL6RA complex towards the GP130 R281Q variant is reduced. GP130-deficient HEK293 cells were transfected with the indicated GP130 variants fused C-terminally to the fluorescent protein YPet and incubated on ice with Hyper-IL-6-Fc (HypIL-6-Fc) or hyper-IL-11-Fc (HypIL-11-Fc) for 30 min. HypIL-6/IL-11-Fc are artificial fusion proteins of IL-6 to soluble IL6RA or IL-11 to soluble IL11RA, respectively, along with a human Fc fragment. Receptor complexes were subsequently isolated by Protein A/G pulldown and analyzed by SDS-PAGE and immunoblotting using the indicated antibodies. Note that YPet-fused GP130 is detected with an anti-GFP antibody. Shown is one representative and the quantification of three independent experiments. *P < 0.05, two-sided Mann–Whitney *U*

We next investigated the structural effects of p.R281Q on GP130/IL11RA/IL-11 signaling. Since published cryo-electron microscopy data suggest a similar hexameric arrangement for the GP130/IL11RA/IL-11 and the GP130/IL6RA/IL-6 complex,^22^ but no crystal structure is currently available for the former, we performed homology modeling. R281 is part of the solvent-excluded GP130/α-receptor interface between the domain D3 of GP130 and domain D3 of IL6RA/IL11RA (Supplementary Fig. 3a, b). Amino acids at this interface show a high evolutionary conservation (Supplementary Fig. 3a, b) and a low genetic variation in human populations as indicated by the minor allele frequencies in the ExAC database (Supplementary Fig. 3c). The solvent-excluded area is larger in the GP130/IL6RA interface as compared to the GP130/IL11RA interface (Supplementary Fig. 3b). Our data suggest that the GP130 Q281 amino acid disrupts all salt bridge and hydrogen bonding to IL11RA, including interactions with IL11RA Y260, T281, and D282, suggesting a specific interaction defect with IL11RA (Fig. 4a–c).

We next analyzed the WT GP130 and GP130 p.R281Q variant in molecular dynamics simulations. Side chain flexibility of R281 was low in both GP130/IL6RA/IL-6 and GP130/IL11RA/IL-11 complexes, consistent with the notion that R281 is engaged in salt bridges and hydrogen bonding in both (Fig. 4d). However, Q281 in the mutant receptor complex displayed a significantly higher degree of freedom (Fig. 4d). Consequently, we observed an increased interdomain distance between GP130 domain D3 and IL11RA D3, but not the IL6RA D3 (Fig. 4e).

Given the importance of GP130 R281 interaction with Y260, D282, and T281 for IL11RA association, we assessed evolutionary conservation of the GP130 D3/α-receptor D3 interface. We observed that amino acids engaged in the solvent-excluded receptor interface, and in particular Arg (R) at the position corresponding to 281 in human GP130, are highly conserved from amphibians to mammals (Supplementary Fig. 3a). The size, position, and residues of the corresponding IL11RA domain D3 receptor interface were also highly conserved. By contrast, IL6RA domain D3 displayed a higher degree of amino acid variation (Supplementary Fig. 3a). These data suggest that IL11RA-containing receptor complexes, in contrast with the IL6RA-containing receptor complexes, are strongly dependent on D3 domain interactions, making them more susceptible to amino acid variations.

In order to analyze cytokine/α-receptor affinity to GP130 variants fused to the fluorescent protein YPet, we made use of Hyper-IL-6-Fc (HypIL-6-Fc)^23^ and Hyper-IL-11-Fc (HypIL-11-Fc).^24^ These are artificial fusion proteins of IL-6 or IL-11 to the soluble ectodomains of IL6RA or IL11RA, respectively, and an additional human Fc-tag, facilitating the isolation of GP130-containing receptor complexes at the plasma membrane (Fig. 4f). We were able to immune-precipitate similar amounts of GP130 as detected by anti-GFP immunoblotting against the YPet-tag of the GP130 variants using HypIL-6-Fc in either WT- or R281Q-expressing cells (Fig. 4f). However, when using HypIL-11-Fc, the amount of precipitated YPet-GP130 was significantly reduced when cells expressed the R281Q variant as compared to the WT (Fig. 4f). Therefore, these in vitro data confirm our in silico predictions that the affinity of interactions between GP130 and IL11RA but not IL6RA is significantly lowered by the p.R281Q variant.

### A mouse model with *Il6st* p.R279Q variant is associated with facial synostosis and exhibits a IL-11 signaling defect

**Fig. 5:**
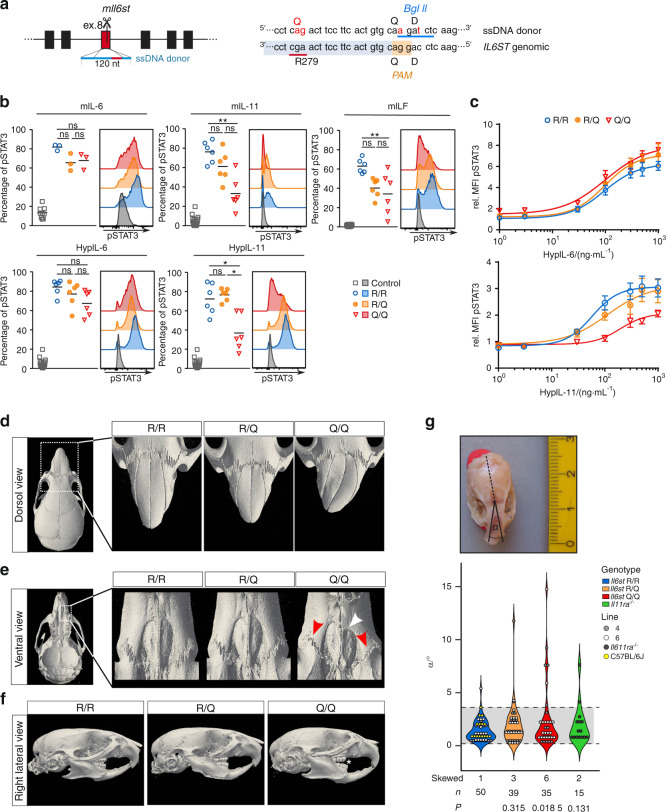
Mice with a homozygous Il6st p.R279Q substitution develop facial synostosis. **a** CRISPR/Cas9-mediated generation of *Il6st p.R279Q* mice. An sgRNA sequence was selected to direct Cas9 cleavage close to the Arg-279-encoding codon. Genome editing was mediated by homology-directed repair with a 120 nt single strand (ss) DNA donor which introduced a c.[835G>A;836A>G] mutation resulting in a p.Arg279Gln exchange. In addition, a c.854G>A silent mutation to destroy the PAM sequence, and a c.857C>T silent mutation to insert a *Bgl*II site were introduced. PAM sequence is highlighted in light orange and sgRNA target sequence in light blue. Nucleotide exchanges are marked in red and the *Bgl*II site is underlined in blue. **b** IL-11 signaling is impaired in *Il6st p.R279Q* homozygous mice. Primary murine skin fibroblasts were stimulated for 15 min with the indicated cytokines and phosphorylation of STAT3 was analyzed by flow cytometry. HypIL-6 and HypIL-11 are artificial fusion proteins of IL-6 and soluble IL6RA or IL-11 and soluble IL11RA respectively. Shown is one representative plot and the quantification of *n* = 3–6 animals/group in technical duplicates in two independent experiments; results from fibroblasts of mouse line 4 and 6 were combined. *P < 0.05, **P < 0.01, Kruskal–Wallis with multiple comparison post-test. **c** Concentration dependency of HypIL-6 and HypIL-11 signaling in primary mouse ear fibroblasts from mice with the indicated genotype. Two independent experiments with three mice/group and concentration. Results from fibroblasts of mouse line 4 and 6 were combined. **d–g** Mice with *Il6st* p.R279Q substitution display craniofacial abnormalities. 3D reconstructions of µCT images of the indicated skull regions of 6-week-old mice are shown. **d** Several homozygous *Il6st* p.R279Q mice (Q/Q) displayed bilateral midface hypoplasia (shortened snout) or sideward deviation of snout growth, and uni- or bilateral abnormalities in naso-frontal sutures. **e** Unilateral premaxillary hypoplasia (white arrowhead) and reduced interdigitation of premaxillary sutures (red arrowheads) in individual homozygous *Il6st* p.R279Q mice (Q/Q). **f** Right lateral views of μCT skull 3D reconstructions indicate class III molar malocclusion (white star) with anterior positioning of lower row of teeth in relation to the upper row in *Il6st* p.R279Q homozygous mice (Q/Q). **g** Several of both heterozygous and homozygous *Il6st* p.R279Q mice (6–24 weeks of age) displayed sideward deviation of snout growth. Deviation from linear growth was determined as α. Gray area and dashed lines indicate variability of wild-type snout growth. The number of mice with skewed snouts and the total number (*n*) of animals are indicated. Phenotype distribution was analyzed by Fisher’s exact test with the indicated number of animals per group

Loss of IL11RA signaling is associated with craniosynostosis and dental abnormalities.^5,14–18^ We hypothesized that impaired IL-11 signaling of the p.R281Q variant accounts for the skeletal abnormalities in the patient. In order to test this hypothesis, we used CRISPR/Cas9 technology to generate two independent lines of mice (lines 4 and 6) that express GP130 p.R279Q corresponding to human GP130 p.R281Q (Figs. 1d and 5a). For this, an sgRNA targeting murine *Il6st* exon 8 and a single-stranded (ss) DNA donor containing substitutions c.[835G>A;836A>G], i.e. the mouse equivalent to the human variant as well as two silent substitutions to destroy the PAM sequence used and to insert a *Bgl*II restriction endonuclease site for genotyping, were injected into one-cell stage mouse embryos (Supplementary Fig. 4a) and implanted into foster mice. Offspring were identified by genotyping (Supplementary Fig. 4b, c).

To analyze the functional consequences of GP130 p.R279Q in mice, we isolated primary skin fibroblasts from WT mice (R/R) and those with the p.R279Q variant (heterozygous R/Q or homozygous Q/Q). We confirmed equal expression and surface localization of the GP130 variants in these cells (Supplementary Fig. 4d). We then stimulated fibroblasts with different cytokines and assessed STAT3 phosphorylation by immunoblotting and flow cytometry. STAT3 phosphorylation was significantly impaired in Q/Q fibroblasts of both mouse lines, when stimulated with IL-11 or Hyper-IL-11, but not when stimulated with IL-6 or Hyper-IL-6 (Fig. 5b, c, Supplementary Fig. 4e). In contrast to the human variant, there was a partial reduction in LIF signaling (Fig. 5b).

We next investigated the phenotype of the animals. Crossing of GP130 p.R279Q heterozygous mice resulted in the expected Mendelian ratio of homozygotes, suggesting that the p.R279Q variant is not lethal (Supplementary Fig. 4f). However, the litter size was significantly reduced when mother animals carried two mutant alleles (Supplementary Fig. 4g). This is reminiscent of the infertility phenotype observed in homozygous female *Il11ra*^*−/−*^ mice.^25^ Flow cytometry analysis of peripheral blood did not show any signs of abnormal hematopoiesis (Supplementary Fig. 4h, i).

In contrast to our individual P^R281Q^, but similar to IL11RA-deficient mice, we did not observe signs of abnormal cranial suture fusion in the skulls of Q/Q mice by µCT analysis (Supplementary Fig. 5a). However, in a proportion of homozygous GP130 p.R279Q mice, we observed macroscopically visible facial malformations (Supplementary Fig. 5b), reminiscent of IL11RA-deficient mice.^5,26^ Microcomputed tomography (µCT) revealed individual mice with sideward deviation of snout growth (Fig. 5d), increased ossification of premaxillary sutures (Fig. 5e), and dental malocclusion (Fig. 5f). Although the penetrance of the phenotype was not complete, the number of mice with facial phenotype was significantly elevated in homozygous Q/Q mice, as assessed by the degree of sideward deviation of snout growth (Fig. 5g). We did not observe signs of increased deposition of bone matrix as trabecular parameters of tibiae were similar in both, R/R and Q/Q mice (Supplementary Fig. 5c).

Taken together, our novel mouse model demonstrates that a selective IL-11 signaling defect of GP130 is sufficient to induce facial synostosis reminiscent of mice with *Il11ra* deficiency.

## Discussion

The shared use of a common signal transducing receptor subunit by cytokines and their specific α-receptor subunits is observed in several cytokine receptor families including the IL-6 family cytokines.^27,28^ Due to the combinatorial nature, variants in the common receptor chain likely affect multiple signaling pathways. We describe a biallelic *IL6ST* variant encoding a GP130 p.R281Q substitution that confers a IL-11-selective signaling defect, while signaling of other IL-6 family members remains intact. Albeit the signaling effects were also observed in patient-derived primary cells, we cannot exclude variations due to the use of non-isogenic primary cells. The craniosynostosis and dental abnormalities seen in the patient with homozygous GP130 p.R281Q are reminiscent of the phenotype of patients with craniosynostosis and dental anomalies caused by *IL11RA* mutations.^5,14–18,29^ In light of the relatively high minor allele frequency of 0.0016 in the South Asian population and the incomplete penetrance in this consanguineous family, a causal relationship between the variant and the phenotype cannot be established with a single case. We therefore generated a mouse model by genomic engineering of the *Il6st* locus, resulting in endogenous expression of a GP130 p.R279Q variant. These mice had facial synostosis mirroring the phenotype developed by homozygous *Il11ra* null mice,^5^ in particular premature ossification of premaxillary sutures, along with abnormalities in nasal bone growth and dental malocclusion. Similar to IL11RA-deficient mice, the facial phenotype of our GP130 p.R279Q mice was not completely penetrant. The apparent reduction in phenotype penetrance in humans and mice may be due to residual IL-11 signal transmission via GP130 p.R279Q seen at the highest concentrations of IL-11. It is likely that high IL-11 concentrations are available in the developing connective tissue explaining the incomplete phenotype of the variant. Similar, in contrast to IL11RA-deficient mice,^6^ we did not observe a significant increase in trabecular bone volume in young GP130 p.R279Q mice. However, at present we cannot exclude that reduction in IL-11 signaling in GP130 p.R279Q mice might reduce loss of bone density in aged mice as has been previously observed in IL11RA-deficient mice.^6^

Environmental factors may additionally influence phenotype penetrance. In humans multiparity, macrosomia,^30,31^ in utero exposure to nicotine^32,33^, and alcohol^34^ have been identified as potential risk factors for the development of craniosynostosis. It will be interesting to see whether changes in the microbiota could also contribute to the variable penetrance of skeletal abnormalities in the context of impaired IL-11 signaling.

Recently, a population of Gli1^+^ cells within sutures of postnatal mice was identified as a major mesenchymal stem cell (MSC) population that gives rise to the osteogenic cells supporting craniofacial bone turnover. Ablation of these MSCs in adult mice resulted in craniofacial suture closure.^35^ Interestingly, suture fusion occurred in mice with incomplete depletion, pinpointing to a certain threshold of MSC number that is needed to maintain suture patency. IL-11 promotes osteoblast differentiation into osteogenic cells in vitro^36–38^ and supports osteoclastogenesis through induction of TNFSF11 (previously termed as RANKL) expression in osteoblasts and also effects on osteoclasts.^6,39^ It is therefore tempting to speculate that IL-11 signaling is responsible for an increase in craniofacial MSC turnover but is not necessary for MSC maintenance. This may explain why the phenotype of *Il11ra*^*−/−*^ and our *Il6st* p.R279Q mice is not 100% penetrant as other signals in some sutures and animals might be sufficient to keep the number of MSCs high enough to prevent suture closure. It may explain why in mice, in contrast to humans, loss of IL-11 signaling does not cause pathological suture fusion of the cranial vault. A similar species-dependent discrepancy in effects of mutant receptor signaling has been observed in *Fgfr3*^*P244R*^-mutant mice that express the *FGFR3*-mutation associated with coronal synostosis in human Muenke syndrome.^40^

In addition to the connective tissue phenotype, the GP130 variant has an effect on reproduction in mice. In mice IL-11 signaling plays an important role for decidualization of endometrial stromal cells. Consequently, *Il11ra*^−/−^ female mice fail to support proper embryonal implantation and are therefore infertile.^25^ Consistent with impaired but not fully blunted IL-11 signaling, we observed a slight reduction in litter size in our GP130 p.R279Q mice, when female mice carried at least one mutant *Il6st* allele. Interestingly, litter size was further decreased if father animals also carried a mutant *Il6st* allele, suggesting additional effects in the embryo itself. It was demonstrated that LIF promotes human blastocyst formation, embryonal stem cell survival^41^ and embryo implantation in mice.^42^ A reduction in LIF signaling in GP130 p.R279Q mice that we observed in primary fibroblasts might therefore account for a decrease in blastocyst survival in these animals. The situation in humans might slightly differ, as females homozygous for inactivating IL11RA variants are able to reproduce and have healthy children.^5,15^ This can be explained by species differences as in mice, decidualization of stromal cells is dependent on the presence of a blastocyst, while in the human female decidualization occurs spontaneously within the late secretory phase of the menstrual cycle.^43^

Our data provide a structural explanation for the cytokine-selective effect of the variant. IL-6 and IL-11 are engaged in a hexameric complex consisting of cytokine, α-receptor and GP130. The receptor complex is formed by contact sites of the cytokine to α-receptor and GP130 (site I, IIa, and III),^44^ and further stabilized by a receptor interface (site IIb) built up between domains D3 of GP130, containing R281, and D3 of the α-receptor. The interface between GP130/IL11RA seems to be smaller than GP130/IL6RA as deduced from our in silico analysis. During evolution, site IIb in both GP130 and IL11RA is highly conserved in all vertebrate animal classes. The amino acid composition of IL6RA domain D3 was more variable suggesting that stability of the IL-6 receptor complex is much less dependent on site IIb than the IL-11 receptor complex. The IL-6 receptor complex might have evolved with a larger surface area at site I, IIa, and III compared to the IL-11 receptor complex. This might explain why the R281Q substitution lowers the affinity of IL-11/IL11RA but not IL-6/IL6RA interactions with GP130. Our analysis underlines that during evolution, GP130 has developed diverse cytokine/receptor interfaces with different characteristics meeting the needs of different cytokines in specific tissues.

Taken together, our analysis of GP130 p.R281Q clearly demonstrate that this variant (I) causes a selective defect in IL-11 signaling due to a decrease in GP130–IL11RA interaction, (II) occurs in an evolutionary conserved region of the protein, (III) is associated with craniosynostosis in one of two human subjects, and (IV) causes facial synostosis in a mouse model. According to previously published guidelines for single-patient genetic variants all criteria, except complete penetrance are fulfilled.^45^ Since the penetrance of the phenotype is incomplete in both, human and mice we only can suggest that based on our data the GP130 p.R281Q variant is causally linked to the observed clinical phenotype.

To the best of our knowledge, the GP130 p.R281Q variant that we identified is the first described cytokine-selective loss-of-function variant in a common signal transducing receptor subunit that affects signal transmission of only one cytokine. Our findings help to understand the fundamental molecular aspects underlying cytokine-selective signaling involving common receptor signaling subunits.

## Materials and methods

### Case studies

The clinical studies were approved by Oxfordshire Research Ethics Committee B (reference C02.143) and London Riverside Research Ethics Committee (reference 09/H0706/20). The proband was enrolled into the craniosynostosis cohort based on referral to a craniofacial unit, with craniosynostosis proven on computed tomography (CT) of the skull.

DNA was extracted from either venous blood collected into EDTA or patient-derived lymphoblastoid cell lines (LCL). All DNA was extracted using the Nucleon Blood and Cell Culture (BACC) DNA extraction kit (Gen-Probe Inc.) according to the manufacturer’s instructions.

Healthy volunteer donors were recruited as part of the Oxford Gastrointestinal Illness Biobank (REC 11/YH/0020) or obtained as leukocyte cones from UK blood donor bank. Informed consent for participation in this study was obtained from healthy donors, patients, or their parents.

### Targeted and Sanger sequencing

We used Fluidigm/Ion Torrent resequencing to screen *IL6ST* in DNA panels from subjects with craniosynostosis who were negative for mutations in the major causative genes as described previously.^19^ Amplicons that failed quality control were reanalyzed using molecular inversion probes.

### Cell culture and cytokine stimulation

HEK293 cells were cultured in Dulbecco’s modified Eagle’s medium (DMEM) supplemented with 10% fetal calf serum (FCS). HEK293 GP130 knockout (KO) cell lines were generated using CRISPR/Cas9 following published protocols.^19,46^ Primary cells and cell lines were stimulated with indicated concentrations of recombinant human IL-6, IL-21, IL-27, OSM, LIF (all Peprotech); IL-10 and IL-11 (both R&D Systems); CT1, CNTF (both Miltenyi Biotec), and CLC (BioLegend).

### Isolation and cultivation of primary mouse skin fibroblasts

For isolation of primary fibroblasts, about 1 cm² of freshly prepared murine ear tissue was incubated for 5 min in 70% ethanol and subsequently handled under sterile conditions. The tissue was air-dried, cut into pieces of a few millimeters, and digested with a mixture of collagenase A (2 mg·mL^−1^), collagenase D (2 mg·mL^−1^), and dispase (4 mg·mL^−1^) diluted in DMEM for 1 h under constant shaking at 37 °C. Cells were isolated by grinding the tissue through cell strainers (70–100 µm), washed once, and then cultured in DMEM + 20% FCS + 1% penicillin/streptomycin under constant conditions at 37 °C, 5% CO_2_, and 96% humidity. At 80%–90% confluency cells were washed with PBS and detached applying 1× Trypsin EDTA for 10 min at 37 °C. Required cell numbers were seeded in either DMEM without serum or DMEM with 20% FCS.

### Flow cytometry of primary mouse skin fibroblasts and peripheral blood

Primary fibroblasts were washed with PBS and incubated with accutase for 3 min at 37 °C. Subsequently still attached cells were gently scraped from the plates and passed through 70 to 100 µm cell strainers. Cell strainers were flushed with PBS and cells collected by centrifugation. Whole blood was collected in heparinized tubes and washed once with PBS prior to staining.

Fibroblasts were stained with anti-gp130-APC-antibody (R&D Systems; clone: #125623) or isotype control for 1 h at room temperature in the dark. Whole-blood cells were stained for 30 min at room temperature in the dark with different combinations of the following antibodies: anti-CD45-BV510 (BioLegend; clone 30-F11), anti-CD3-FITC (BioLegend; clone145-2C11), anti-CD19-APC (BioLegend; clone HiB19), anti-CD4-APC/Cy7 (BioLegend; clone RM4-5), anti-CD8-PE/Cy7 (BioLegend; clone 53–6.7), anti-Ly6G-FITC (BioLegend; clone RB6-8C5), anti-Ly6C-APC/Cy7 (BioLegend; clone HK1.4), anti-CD115-PE/Cy7 (BioLegend; clone AFS98). Stained whole-blood cells were subsequently incubated with RBC Lysis and Fixation Solution (BioLegend) for 15 min at 37 °C and washed twice with PBS. Samples were acquired in FACS buffer [1% BSA in PBS] on a BD FACS Canto II flow cytometry system and analyzed with FlowJo Software (Version 10.5.3, Tree Star).

### STAT3 and STAT1 phosphorylation assays

Unless indicated otherwise, phosphorylation of STAT1 or STAT3 transcription factors was assessed as described by Schwerd et al.^19^ by phosflow, immunofluorescence and confocal microscopy or luciferase STAT3 reporter assay. Phosphorylation assays were mainly performed in parallel to evaluation of patient P^N404Y^ described by Schwerd et al.^19^ and results from healthy donors or WT controls are duplicated to ensure perfect comparability.

In addition to phosflow assessment, STAT3 phosphorylation in murine cells was analyzed by SDS-PAGE and immunoblotting. In brief, 24 h prior to analysis primary fibroblasts were seeded in six-well plates. The next day cells were serum starved for 4 h and stimulated with cytokines at the indicated concentrations for 10 min at room temperature. Stimulation was immediately followed by cell lysis for 15 min on ice with RIPA buffer [50 mmol·L^−1^ Tris, 150 mmol·L^−1^ NaCl, 0.1% SDS, 0.3% sodium deoxycholate, 1% Triton X-100] with freshly added protease and phosphatase inhibitors. Samples were separated by SDS-PAGE on 10% bis-tris-gels and subjected to immunoblotting using primary antibodies anti-gp130 (R&D Systems; clone: #125623), anti-STAT3 (Cell Signaling; clone 124H6), anti-phospho-STAT3 (Cell Signaling; clone: D3A7), anti-beta-actin (Sigma Aldrich; clone AC-15). Signal intensity was determined by densitometry using ImageJ (version 1.52n).

### Lentivirus production and IL11RA overexpression in CD4^+^ memory T cells

The empty vector pCDH-EF1-MCS-T2A-copGFP (CD526A-1) and the vector carrying human *IL11RA* transcript variant 3 (NM_001142784.2) for ectopic expression were purchased from SBI Systems Biosciences.

Lentiviral particles were produced by transiently transfecting HEK293 cells with the above described transfer vectors together with the ViraPower™ lentiviral packaging mix (Invitrogen) in 150 mm cell culture dishes (Corning). Briefly, HEK293 cells were transfected with a cocktail of transfer vector and packaging mix in Opti-MEM (Gibco), using Lipofectamine® 2000 (Thermo Fisher) as a transfecting agent according to the manufacturer’s instructions. Culture supernatants containing viral particles were harvested at 72 h post-transfection and titers were determined by limiting dilution on HEK293 cells.

Resting memory CD4^+^ T cell lines were transduced by spin infection (60 min; 800 *g*; 32 °C) on anti-CD3 (5 μg·mL^−1^; Biolegend; clone: OKT3) and anti-CD28 (1 μg·mL^−1^; BD Biosciences; clone CD28.2) coated 24-well plates (1 × 10^6^ cells/well) in the presence of 5 μg·mL^−1^ polybrene. The medium was then replaced and cells were cultured in IL-2-containing (500 U·mL^−1^) medium. After 48 h of culture, cells were transferred to uncoated plates. Following a minimum of 7 days of culture, expanded T cells were starved overnight in IL-2-free medium, washed extensively, and analyzed for IL-10 and IL-11 responsiveness after 30 min of stimulation by intracellular staining for phosphorylated STAT1 and STAT3. To exclude non-transduced T cells from the analysis gating was performed on the GFP^+^ population. Flow cytometry data were analyzed with FlowJo (Version, Tree Star).

### Receptor complex isolation and immunoblotting

GP130-deficient HEK293 cells were transiently transfected with plasmids encoding for GP130 WT-YPet or GP130 R281Q-YPet variant using linear polyethyleneimine Max (Polysciences #24765) as transfection agent. Twenty-four hours post-transfection cells were starved overnight in DMEM with 0.5% FCS followed by incubation on ice for 30 min and subsequent stimulation with recombinant Hyper-IL-6-Fc or Hyper-IL-11-Fc for 30 min on ice. Cells were lysed at 4 °C in RIPA buffer (50 mmol·L^−1^ Tris, 150 mmol·L^−1^ NaCl, 0.1% SDS, 0.3% sodium deoxycholate, 1% Triton X-100) supplemented with protease and phosphatase inhibitors. Lysates were cleared by centrifugation at 12 000 *g* for 15 min.

Lysates for immunoprecipitates were incubated with Protein A/G beads (Millipore) for 1 h at 4 °C. Subsequently washed three times in RIPA Buffer supplemented with protease and phosphatase inhibitors. Beads were incubated for 5 min at 90 °C in 2× reducing Laemmli Buffer.

All samples were analyzed by 10% bis-tris SDS-PAGE and immunoblotting. The following antibodies were used: anti-GFP (Roche, 11814460001), anti-beta-actin (clone: AC-15) and anti-IgG-Fc (R&D Systems, Cat.No. G-102-C). Secondary antibodies were anti-mouse-HRP or anti-rabbit-HRP (both Dianova).

### Homology modeling and molecular dynamics (MD) simulations

Due to the unavailability of IL11RA structure coordinate files, a model was generated using the structure coordinates of IL6RA (pdb entry 1P9M) as template and amino acids 112–320 of the IL11RA sequence deposited in UniProt Q14626 with matching alignment to the IL6RA. Modeling was performed using the MODELLER interface of the UCSF Chimera package.^47^ WT R281 in GP130 was exchanged to Q and the corresponding rotamer was introduced searching the Dunbrack library.^48^ The rotamer with the highest χ-angle probability score was chosen.

Molecular dynamics calculations were performed on trimeric complexes either consisting of GP130/IL6RA/IL-6 or of GP130/IL11RA/IL-11 using AmberTools17 (ref. ^49^) compiled for parallel computing on 32 cores. IL-11 (4MHL.pdb) was fitted into the complex by superimposing it with IL-6 with the tool MatchMaker of UCSF Chimera.^50^ Initially, the protein force field ff14SB was applied to the structure models and a solvate box was generated using the tip3p water model.^51^ For the GP130 p.R281Q variant, overall charge was neutralized using Na^+^ ions. To solve possible high-energy states, 2 000 steps of minimization were performed prior to MD simulation. For the MD simulation, the system was heated to 300 K over 100 ps and hold at constant volume and temperature conditions (NVT) for additional 20 ps. Subsequently, parameters were changed to 1 bar constant pressure and 300 K constant temperature (NPT) to resemble laboratory conditions and structural dynamics were analyzed for 1 ns in the NPT ensemble. All calculations were performed by parallel computing on a NEC HPC Linux-Cluster at the CAU University Computer Center.

Trajectory files from the MD simulations were analyzed using UCSF Chimera. The center of mass for GP130 D3 was defined of residues 198–298 and residues 215–317 for the IL11RA. To analyze rotamer flexibility throughout the 1 ns simulation every 0.1 ns PDBs were saved and subsequently superimposed using the MatchMaker tool of UCSF Chimera. Root mean square deviation (RMSD) of arginine or glutamine, respectively, was calculated as mean RMSD throughout the simulation relative to positions of arginine/glutamine at 0 ns.

The mean buried area between GP130 D3 and the alpha receptor D3 was calculated from solvent-excluded surfaces from ten equally distributed time points of the 1 ns MD simulations of the IL-6 and the IL-11 complex, respectively. Information about highly contributing amino acids were extracted at the last frame of the simulations.

### Analysis of evolutionary conservation

Conservational scores were calculated with the AL2CO algorithm in UCSF Chimera on the basis of multiple sequence alignments. For conservation per class, 170 sequences for GP130 were retrieved from the NCBI protein database and subjected to multiples sequence alignment using Clustal Omega.^52^ Alpha receptor sequences were obtained by blast search with a threshold of e^−4^ in the RefSeq-Database against the full-length alpha receptors (IL6RA: UniProt P40887, IL11RA: UniProt Q14626) using NCBI Protein BLAST. We only selected sequences annotated for IL6RA or IL11RA from species that also displayed an annotated database entry for GP130 and subjected them to multiples sequence alignments using Clustal Omega (Supplementary Table 2). AL2CO scores were mapped on a representative structure per class obtained by homology modeling of a class-specific sequence against the human ortholog. In the case of mammals, the human structures were used. Amino acids corresponding to pos. 281 in human GP130, as well as amino acids corresponding to pos. 269, 281, 282 in IL11RA, and the corresponding amino acids in IL6RA were labeled in the structures. Amino acids contributing to the buried interface were encircled.

Data on mutational hotspots in GP130, IL6RA, and IL11RA were obtained from the Exome Aggregation Consortium (ExAC). Results were converted into a format readable for mapping with UCSF Chimera and subsequently mapped onto the structures of human GP130 D3, IL6RA D3, and modeled IL11RA D3.

### Mouse model generation

The sgRNA sequence targeting exon 8 of murine *Il6st* was designed using the CRISPOR Program.^53^ The template for transcription was derived by PCR using Q5-Polymerase (Biolabs). Transcription was performed using the HiScribeT7 kit (Biolabs, E20140S) with subsequent purification of the transcript with the MEGAClearTM kit (Fisher Scientific, AM1908), both according to the manufacturer’s instructions.

One-cell stage embryos derived from superovulated C57BL/6JUke mice were injected using 10 ng·μL^−1^ sgRNA, 20 ng single stranded repair template (Sigma) introducing NM_010560:c.[835G>A;836A>G] for the p.R279Q substitution and further silent mutations for a *Bgl*II restriction site and a degenerated PAM sequence. We selected the CAG codon for glutamine, as the CAA codon is infrequently used in mice. The repair template (CTCCAGTAGCCCTTCCCACTGTCCTTAATGGACCGGATCCTAAACACATATTCTGTAAAAGGCTTGAGaTCtTGCACAGTGAAGGAAGTctGAGGAGACATTGTATCTTCAAGAGGGACC) was transfected jointly with 50 ng·μL^−1^ Cas9 protein (IDT). Embryos were implanted into F1 foster mothers (C57BL6 × CBA) and the resulting offspring was analyzed by PCR using (Il6st-F: GGT CTG GTT CTT TAA GAC AGG CTC TC, Il6st-rev: CAC CAC TTT TAC GTA TGT CTT CGT ATG TG) and *Bgl*II digestion. Correct integration of the repair construct was verified by sequence analysis. Two independent lineages (termed lines 4 and 6 throughout the manuscript) with *Il6st* c.[835G>A;836A>G] mutation (p.R279Q) were obtained and further bred at the CAU Animal Facility. All experiments were performed in accordance with the local guidelines for animal care and protection.

Il11ra-deficient mice^54^ were on a C57BL/6 background and maintained as previously described.^25^

### Analysis of craniofacial and skeletal phenotype in mice

Skulls were collected from WT, heterozygous p.R279Q mutant, homozygous p.R279Q mutant littermates of mouse lines 4 and 6, as well as mice and stripped of flesh and tendons. Skulls were fixed in 10% buffered formalin at 4 °C for 1 to 2 days and subsequently stored in PBS containing 0.05% sodium azide at 4 °C. Images of cleaned and fixed skulls were taken and lateral twisting of snouts further analyzed. The angle between snout tip and sagittal suture was determined using ImageJ Software (Version 1.52n).

During macroscopic phenotype assessment, skulls were determined as phenotypically aberrant if one of the following criteria were met: sideward deviation of snout, shortening of snout, or downward deviation of snout.

Skulls from three animals and long bones from eight to ten animals per genotype were embedded in 1% agarose in ddH_2_O for µCT analysis. µCT scans were performed at the Molecular Imaging North Competence Center (MOIN CC), Department of Radiology and Neuroradiology, University Medical Center Schleswig-Holstein using a vivaCT 40 (70 kVp, 114 μA, 300 ms integration time, 1 000 projections on 180° 2048 CCD detector array, cone-beam reconstruction, ScancoMedical). All scans were done at an isotropic voxel size of 15.6 μm. Images were further analyzed using ImageJ (Version 1.52n) and ParaView (Version 5.6.0, Kitware) software. Morphometric analysis of tibiae was performed ex-vivo using a VivaCT 80 (Scanco AB, Brüttisellen, Switzerland) micro-CT scanner with 15.6 µm isotropic voxel size (70kBp, 114 µA, 31.9 mm FOV, 300 ms integration time, software binning: 1.5, no HW binning, 1 000 projections/180°, standard reconstruction with beam hardening correction, bone calibration in mgHA·cm^–3^). For segmentation and quantification of parameters, the manufacturer´s software was used. For trabecular measurements a volume of interest (VOI) of 2 mm (126 slices) axial length was selected starting 0.3 mm (20 slices) below the epiphyseal plate. Trabecular bone was contoured automatically in the diaphyseal area, but partially by manually drawing 2D regions of interest (ROIs) every 5–10 slice with geometric morphing approaching the thin cortex and more complex endosteal envelope near the proximal part of the tibiae (Scanco uct_evaluate V6.3–5). The images were binarized using a threshold of 250 mgHA·cm^−^³, resulting in a mask solely with bone and background voxels. Trabecular bone volume density (bone volume (BV)/total volume (TV)), trabecular number (Tb.N), trabecular thickness (Tb.Th), and trabecular separation (Tb.Sp) were calculated (IPL V5.15).

### Protein sequence alignment

Multiple sequences were aligned using ClustalW2.^55^ Data were obtained from the National Center for Biotechnology Information (NCBI). Sequence alignment is based on the following accession numbers: NP_001106976.1, NP_001124412.1, NP_990202.1, NP_034690.3, NP_002175.2, NP_004834.1, NP_000751.1, NP_002301.1, NP_003990.1, NP_005526.1 and NP_001550.1.

### Statistical analysis

Results were analyzed with GraphPad Prism version 5.00 (GraphPad software, Inc., San Diego, CA) or Rstudio (version 1.2.1335). Significance was determined by two-sided Mann–Whitney *U* test, one-way ANOVA, or Kruskal–Wallis with multiple comparisons post-test. Distribution of extreme snout deformation was analyzed using Fisher’s exact test. *P* values below 0.05 were considered as significant.

### Online resources/URLs

The following online data sources have been accessed:

1 000 Genomes. http://www.1000genomes.org

dbSNP. http://www.ncbi.nlm.nih.gov/SNP

GenBank. http://www.ncbi.nlm.nih.gov

gnomAD. http://gnomad.broadinstitute.org/

PolyPhen. http://genetics.bwh.harvard.edu/cgi-bin/pph

SIFT. http://sift.jcvi.org

STRING. http://string-db.org

ExAC browser. http://exac.broadinstitute.org

GDI-server. http://pec630.rockefeller.edu:8080/GDI/resultGeneOnly.jsp

COSMIC. http://cancer.sanger.ac.uk/cosmic/gene/analysis?ln=IL6ST

Blast. https://blast.ncbi.nlm.nih.gov/Blast.cgi

## Supplementary information

Supplemental Material
