# Supplementary material for: A variant in IL6ST with a selective IL-11 signaling defect in human and mouse
Source: Bone Res. 2020 Jun 11;8:24. doi: 10.1038/s41413-020-0098-z (PMC7289831; doi:10.1038/s41413-020-0098-z)
Supplement: Supplementary file 1 — Supplemental Material [file 41413_2020_98_MOESM1_ESM.docx]

SUPPLEMENTARY INFORMATION FOR

**A variant in *IL6ST* with a selective IL-11 signaling defect in human and mouse**

Tobias Schwerd, Freia Krause, Stephen R. F. Twigg, Dominik Aschenbrenner, Yin-Huai Chen, Uwe Borgmeyer, Miryam Müller, Santiago Manrique, Neele Schumacher, Steven A. Wall, Jonathan Jung, Timo Damm, Claus-Christian Glüer^8^, Jürgen Scheller, Stefan Rose-John, E. Yvonne Jones, Arian Laurence, Andrew O. M. Wilkie, Dirk Schmidt-Arras, Holm H. Uhlig*

* Correspondence: Holm H. Uhlig (holm.uhlig@ndm.ox.ac.uk),

**The PDF includes**

| Supplementary Figures with Legends | Fig.S1-S5 |
| --- | --- |
| Supplementary Table | Table S1-2 |
| Supplementary References |  |

**Supplementary Fig. 1**


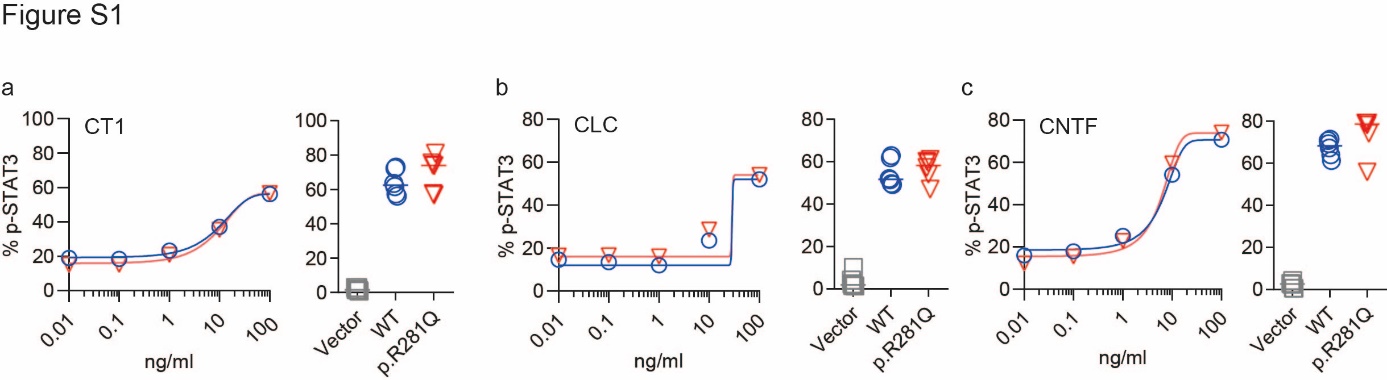


**Supplementary Figure 1. The GP130 p.R281Q substitution causes defective signaling of IL‑11, but not CT1, CLC and CNTF.**

**(a-c)** HEK293 GP130-KO cells were transfected with empty vector control or plasmids encoding GP130 wild‑type (WT) or the patient variant p.R281Q. Cells were stimulated with indicated concentrations of CT1 **(a)**, CLC **(b)** or CNTF **(c)** for 15 minutes and analyzed for STAT3 phosphorylation (pSTAT3) by phosflow. For assessment of CT1 signaling, cells were co-transfected with plasmids encoding LIFR. For assessment of CLC and CNTF signaling, cells were co-transfected with plasmids encoding LIFR and CNTFR. Co-transfection with GFP allowed gating on successfully transfected cells. Representative titration curves (on left in each panel) are shown for each ligand and are representative of one experiment. Curve fitted by non-linear regression. Quantification (on right in each panel) is based on 3 independent experiments per cytokine with duplicates per experiment at maximal concentration.

**Supplementary Fig. 2**


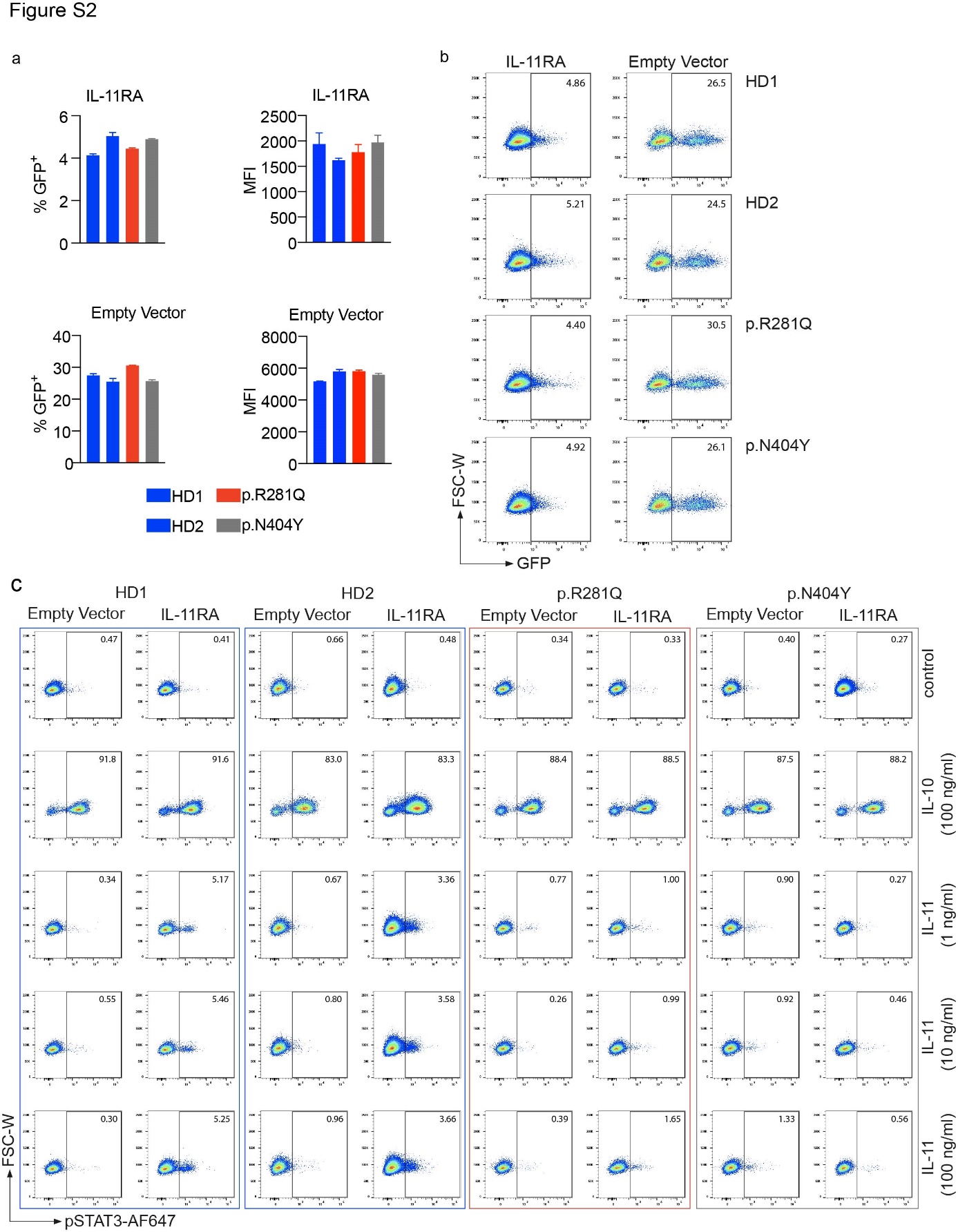


**Supplementary Figure 2.**  **Ectopic expression of IL-11RA in CD4+ T cells**.

**(a)** Summary of frequencies and mean fluorescence intensity (MFI) of empty vector or IL‑11RA T2A-linked GFP expressing CD4+ T cells post lentiviral transduction. **(b)** Dot plot presentation of frequencies of GFP+ cells according to (A). **(c)** Example plots showing the flow cytometry analysis of STAT3 phosphorylation in diverse lentivirus-transduced CD4+ T cells across stimulation conditions gated on single live cells.

**Supplementary Fig. 3**


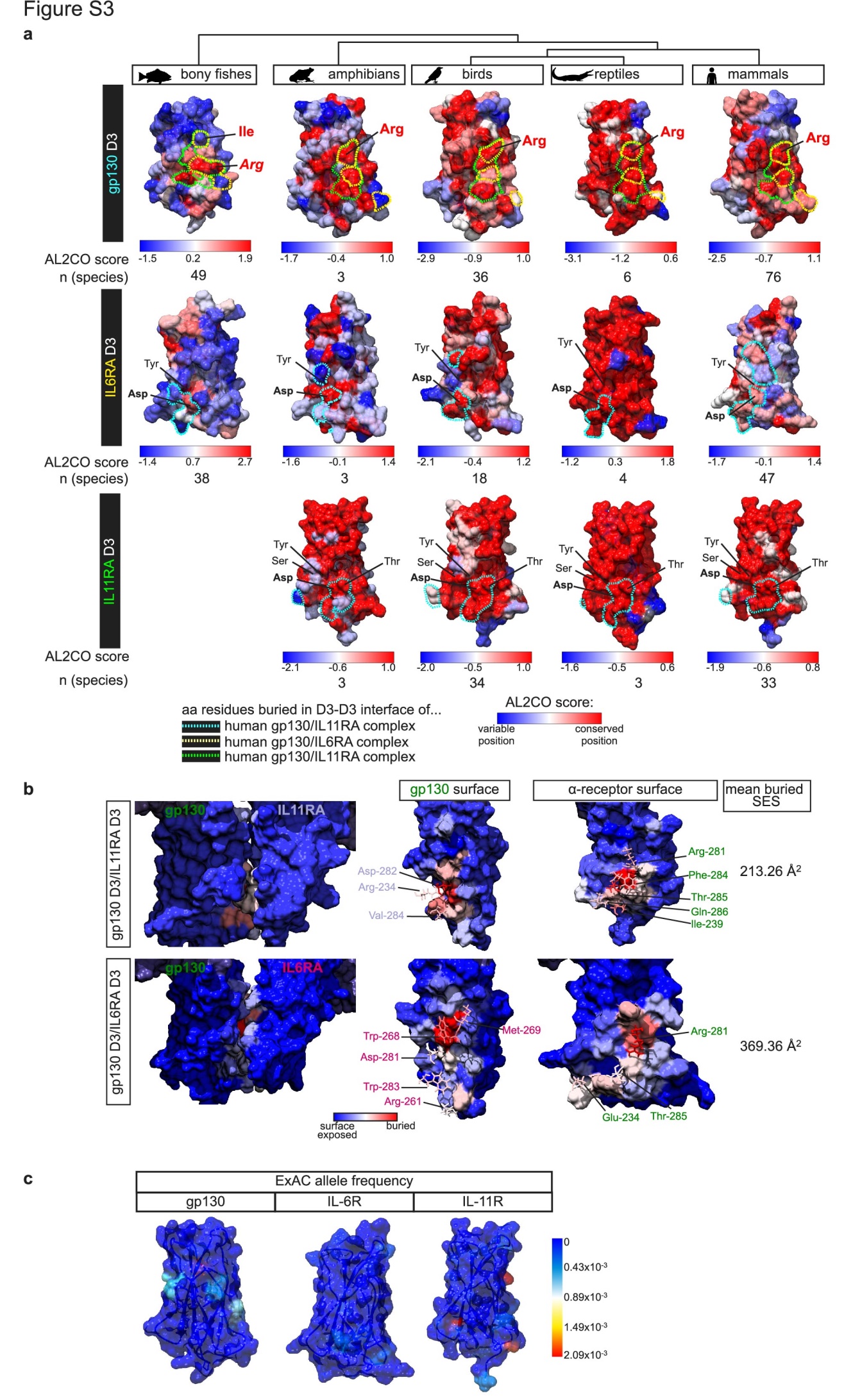


**Supplementary Figure 3. Stability of the GP130/IL11RA/IL-11 complex is strongly dependent on its D3-D3 interactions.**

**(a)** The GP130/IL11RA/IL-11 D3-D3 interface is evolutionary highly conserved. Multiple sequence alignments of either GP130, IL6RA and IL11RA sequences from the indicated number of species per animal class, respectively, were used to calculate the Alignment to algorithm score (AL2CO), that is based on estimated amino acids frequencies at each position. The conservational score was mapped on representative structures of domain D3 of either GP130, IL6RA or IL11RA. For mammalian GP130 and IL6RA D3 structures from 1P9M.pdb were used. All remaining structures were generated by homology modelling based on 1P9M.pdb. Amino acids buried in the D3-D3 interface in the human receptor complex are encircled by dotted lines. The invariant Arg corresponding to position 281 in human GP130 is highlighted. Note that in bony fishes this functionality might be replaced by Arg corresponding to Thr-285 in human GP130. Amino acids interacting with GP130 invariant Arg are labeled within IL6RA and IL11RA D3 structures. An invariant Asp buried within the D3-D3 interface is labeled in bold letters. Conservational score for bony fish IL11RA could not be determined as there was only one annotated bony fish IL11RA sequence available in the NCBI protein database (Supplementary Table 2). **(b)**D3-D3 interface in the GP130/IL6RA/IL-6 complex is increased compared to the GP130/IL11RA/IL-11 complex. Buried solvent-excluded surface in the GP130/IL-6/IL6RA and the GP130/IL11RA/IL-11 D3-D3 interfaces. Contribution of each amino acid to the buried area was calculated at the last frame of the 1 ns MD simulation of the corresponding trimeric GP130 complex. Mean solvent-excluded surface was calculated from measurements every 0.1 ns during the 1 ns MD simulations. ("Blue - White - Red" indicates “surface exposed - mildly buried- heavily buried" on a structure dependent relative scale.) **(c)**Frequency of nucleotide variations obtained from ExAC database were structurally mapped on D3 domains of GP130, IL6RA and IL11RA. Structure of GP130 and IL6RA were obtained from 1P9M.pdb. Structure of IL11RA was obtained by homology modelling using 1P9M.pdb.

**Supplementary Fig. 4**


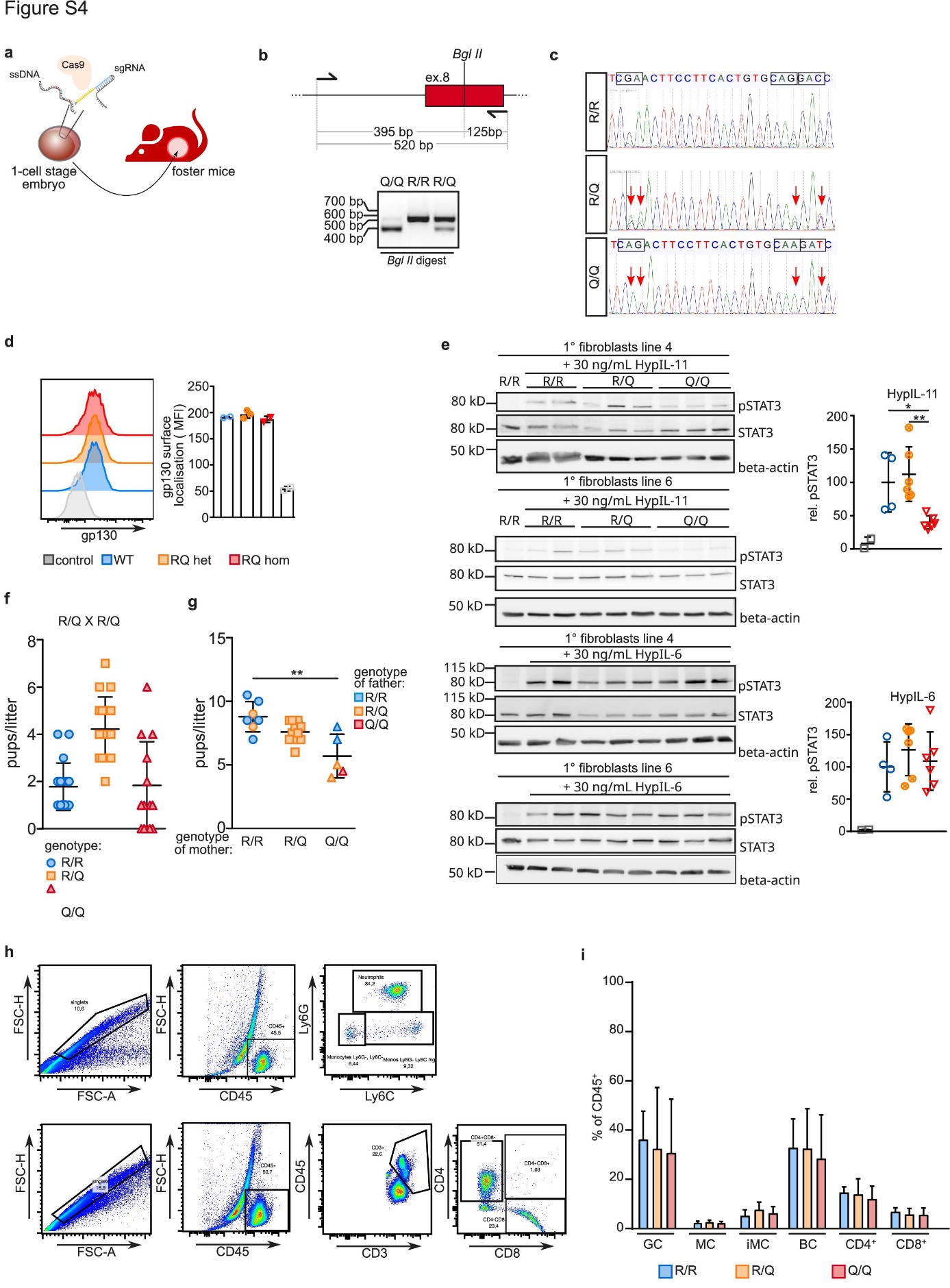


**Supplementary Figure 4. Generation of mice with a *Il6st* p.R279Q substitution corresponding to human *IL6ST* p.R281Q.**

**(a)** Murine C57BL/6JUke 1-cell stage embryos were microinjected with Cas9 nuclease, a sgRNA targeting *Il6st* exon 8 and a 120bp single strand DNA donor (repair template). Microinjected embryos were subsequently implanted into C57BL/6 x CBA foster mice. **(b)** Genotyping strategy of *Il6st* p.R279Q mice. PCR amplification strategy of exon 8. Amplicons from recombined alleles are BglII-sensitive, resulting in smaller fragments, while WT amplicons are detectable at 520bp. R/R: WT/WT, R/Q: WT/p.R279Q, Q/Q: p.R279Q/p.R279Q. **(c)** Sequencing of an *Il6st* ex.8 PCR fragment confirms precise genome editing. Codons with nucleotide exchanges are marked with black boxes. Red arrows indicate nucleotide changes in the electropherogram. **(d)** Surface localisation of GP130 on primary murine skin fibroblasts with the indicated genotype as determined by flow cytometry. Shown is one representative plot and the quantification of 2-3 mice/group. Results from mouse lines 4 and 6 were combined. **(e)** IL-11 but not IL-6 signaling is impaired in primary fibroblasts with *Il6st* p.R279Q genotype. Primary murine skin fibroblasts from the indicated mouse lines were stimulated with 30 ng/ml of the indicated Hyper-cytokines and STAT3 phosphorylation was analyzed by immunoblotting. pSTAT3 from three independent experiments was quantitated by densitometry using ImageJ. Every dot represents fibroblasts from an individual mouse. n=2-6 animals/group, results from mouse lines 4 and 6 were combined. *P<0.05, **P<0.01, one-way ANOVA with Tukey’s multiple comparisons post-test. **(f)** Genotype distribution per litter in heterozygous (R/Q x R/Q) breedings. n=13 litters from 9 independent breeding pairs were analysed and the results from mouse lines 4 and 6 were combined. **(g)** Litter size depending on parents’ genotype. n=28 litters from 14 independent breeding pairs analysed, results from mouse lines 4 and 6 were combined. **P<0.01, Kruskal-Wallis with multiple comparison post-test **(h, i)** Peripheral blood composition of *Il6st* p.R279Q mice is unaltered. Gating strategy (h) and relative numbers of indicated leukocyte populations (i). GC: granulocyte, MC: monocyte, iMC: inflammatory monocyte; BC: B cells, CD4^+^, CD8^+^: T cell subpopulations, n=2-8 animals/group, results are shown for mouse line 6.

**Supplementary Fig. 5**


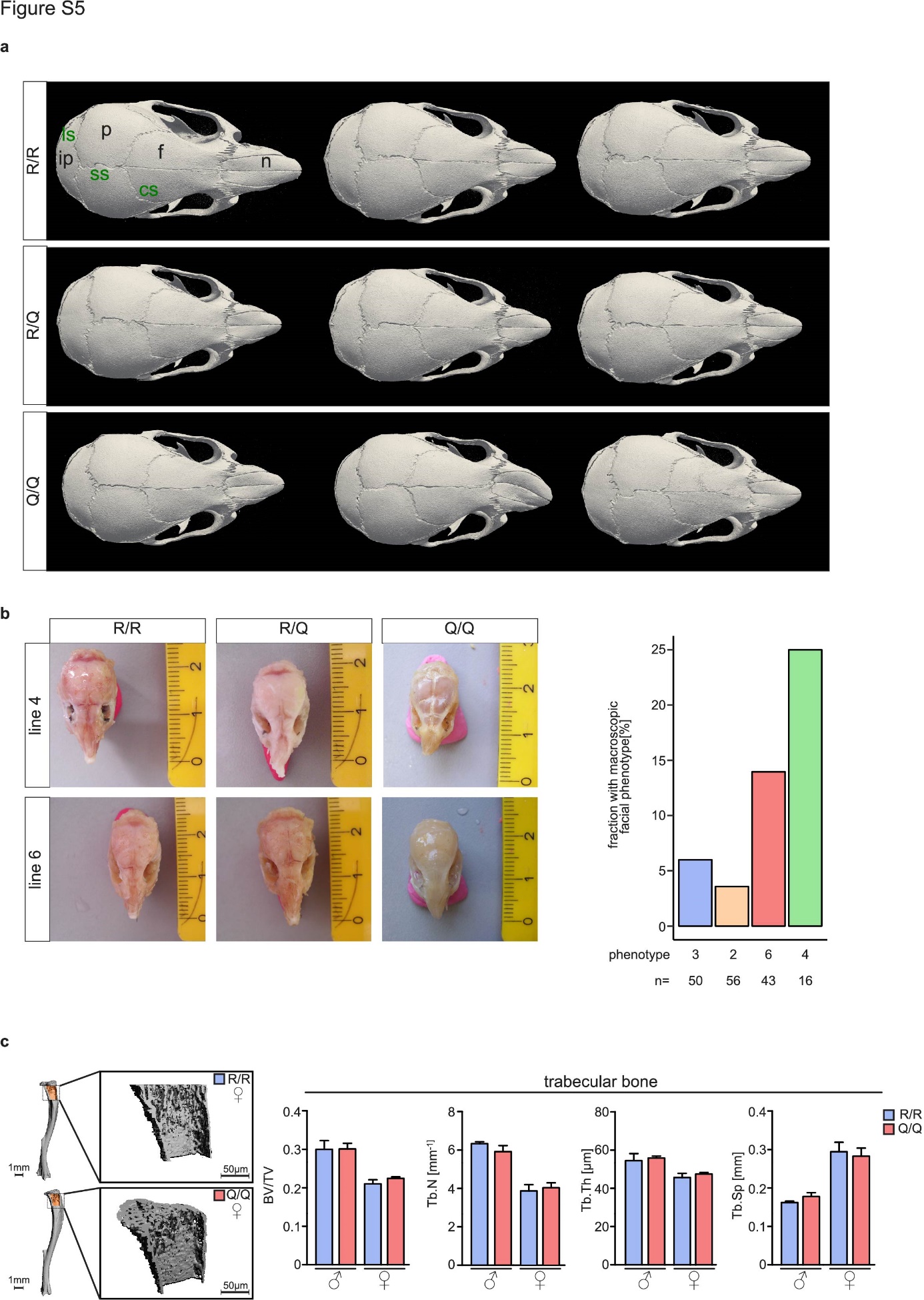


**Supplementary Figure 5. Mice with homozygous GP130 p.R279Q substitution develop facial synostosis.**

**(a)** Top view of μCT skull 3D reconstructions, which display no signs of premature fusion of lambdoid suture (ls), sagittal suture (ss) or coronal suture (cs) in 6 week-old *Il6st* mutant mice. ip: interparietal bone, p: parietal bones, f: frontal bones, n: nasal bones. **(b)** Macroscopically visible facial phenotype in *Il6st* mutant mice (left). The number of mice with facial phenotype (including *Il11ra*^-/-^ mutants) and the total number (n) of animals are indicated (right). **(c)** Representative 3D reconstruction of trabecular (upper panel) and cortical (lower panel) bone µCT images. Morphometric quantification of trabecular and cortical bone parameters of tibiae from mice with the indicated genotype. Bone volume (BV)/total volume (TV): bone volume density; Tb.N: trabecular number; Tb.Th: trabecular thickness; Tb.Sp: trabecular separation.

**Supplementary Table 1**

| **Clinical spectrum of**  **GP130 deficiency** | | **Autosomal recessive**  **Craniosynostosis (this report)** | **Autosomal recessive Hyper-IgE syndrome ^1,2^** | **Autosomal dominant ** Hyper-IgE syndrome** | **Autosomal recessive Stüve-Wiedemann snydrome (SWS) ^3^** |
| --- | --- | --- | --- | --- | --- |
| **Organ Affected** | **Phenotype and**  **number of patients reported** | N = 1  *n=2 (genotype) | N = 2 | N = 12 | N = 6 |
| Growth | Short stature (height < 3rd percentile) | ✓ | ✓ | n.a. | ✓ |
| Skin | Eczema | 🗶 | ✓ | ✓ | ✓ |
| Head and neck | Craniosynostosis | ✓ | ✓ | 🗶 | 🗶 |
|  | Retained teeth | ✓ | ✓ | ✓ | 🗶 |
| Bones and joints | Scoliosis (30° thoracolumbar curve) | 🗶 | ✓ | ✓ | ✓ |
|  | Flexion contracture of the small joints of the hands and elbows | 🗶 | ✓ | n.a. | n.a. |
|  | Severe bowing of long bones | 🗶 | 🗶 | 🗶 | ✓ |
|  | Hip dislocation | 🗶 | ✓ | 🗶 | n.a. |
|  | Destructive arthropathy | 🗶 | ✓ | n.a. | 🗶 |
| Infections | Acute phase response | ✓ | 🗶 | ✓ | 🗶 |
|  | Recurrent upper respiratory tract infections | 🗶 | ✓ | ✓ | 🗶 |
|  | Recurrent otitis media | 🗶 | ✓ | ✓ | 🗶 |
|  | Recurrent pneumonia, empyema and pneumatocele | 🗶 | ✓ | ✓ | 🗶 |
|  | Bilateral keratitis | 🗶 | ✓ | 🗶 | 🗶 |
|  | Fungal infections | 🗶 | ✓ | ✓ | 🗶 |
| Nervous system | Mental motor retardation | 🗶 | ✓ | 🗶 | ✓ |
| Others | Death in utero, early neonatal death | 🗶 | 🗶 | 🗶 | ✓ |

**Supplementary Table 1. Summary of phenotypic spectrum of patients with genetic defects in *IL6ST.***

Table adapted from Shahin et al. (Shahin T, et al., Haematologica 2019). *Please note that the mother of P^R281Q^ was homozygous for the same variant but without any history of craniofacial or severe tooth abnormalities. ** Beziat et al. 2020 J Exp Med in press. Abbreviations: n.a., not assessed or not available.

**Supplementary Table 2**

|  | **bony fishes** | **amphibians** | **birds** | **reptiles** | **mammals** |
| --- | --- | --- | --- | --- | --- |
| **GP130** | *Danio rerio* | *Xenopus laevis* | *Anas platyrhynchos* | *Chelonia mydas* | *Homo sapiens* |
|  | NP_001106976.1 | NP_001124412.1 | XP_005019925.3 | XP_007053933.1 | AAI17403.1 |
| **IL6RA** | *Labrus bergylta* | *Xenopus laevis* | *Haliaeetus leucocephalus* | *Chrysemys picta bellii* | *Homo sapiens* |
|  | XP_020497586.1 | XP_018087196.1 | XP_010562477.1 | XP_023964311.1 | NP_000556.1 |
| **IL11RA** | *Lepisosteus oculatus* | *Xenopus tropicalis* | *Corvus brachyrhynchos* | *Chelonia mydas* | *Homo sapiens* |
|  | XP_015195730.1 | NP_001093752.1 | XP_017602130.1 | XP_007055975.1 | NP_001136256.1 |

**Supplementary Table 1: Protein sequences representative for the indicated vertebrate classes used for homology modelling.**

Species name and NCBI accession number is indicated.

**Supplementary References**

1 Schwerd, T. *et al.* A biallelic mutation in IL6ST encoding the GP130 co-receptor causes immunodeficiency and craniosynostosis. *J Exp Med* **214**, 2547-2562, doi:10.1084/jem.20161810 (2017).

2 Shahin, T. *et al.* Selective loss of function variants in IL6ST cause Hyper-IgE syndrome with distinct impairments of T-cell phenotype and function. *Haematologica* **104**, 609-621, doi:10.3324/haematol.2018.194233 (2019).

3 Chen, Y. H. *et al.* Absence of GP130 cytokine receptor signaling causes extended Stuve-Wiedemann syndrome. *J Exp Med* **217**, doi:10.1084/jem.20191306 (2020).
